# Hospital Expenditure at the End-of-Life: A Time-to-Death Approach

**DOI:** 10.34172/ijhpm.2020.88

**Published:** 2020-06-20

**Authors:** Vahid Alipour, Abolghasem Pourreza, Majid Kosheshi, Hassan Heydari, Sara Emamgholipour Sefiddashti

**Affiliations:** ^1^Health Management and Economics Research Center, Iran University of Medical Sciences, Tehran, Iran.; ^2^Department of Health Education and Promotion, School of Public Health, Tehran University of Medical Sciences, Tehran, Iran.; ^3^Department of Demography, Social Science Faculty, Tehran University, Tehran, Iran.; ^4^Department of Economic Sciences, School of Management and Economics, Tarbiat Modares University, Tehran, Iran.; ^5^Department of Health Management and Economics, School of Public Health, Tehran University of Medical Sciences, Tehran, Iran.

**Keywords:** Hospital Expenditure, Time-To-Death, Aging, Iran

## Abstract

**Background:** In recent years the use of time to death (TTD) variables in the modeling of individual health expenditures has been of interest to health economics researchers. The aim of this study was to investigate the effect of age and TTD on hospital inpatient expenditure (HIE).

**Methods:** We used a claims database from Iran Health Insurance Organization of Tehran city that includes considerable proportion of Tehran residents and contains information on insured individuals’ HIE. We included HIE of all insured decedents (30 to 90 years old) who died during March 2013 and March 2014 (n=1018). No sampling was required. According to the decedents’ date of death, we extracted their last 24 months HIE. The period of time March 30, 2011 until March 30, 2014 (3 years) was used to guarantee a full 24 months of observations for decedents. A two-part econometric model was employed to investigate the effect of age, TTD, and some demographic variables on probability and conditional amount of individuals’ hospital expenditure. Stata software (version 16.0) was used for data processing and analysis.

**Results:** Our results demonstrated that the month-based TTDs especially near months before death of decedents (TTD1 to TTD10) significantly affected both probability and conditional amount of HIE. One month before death incurred more HIE than the rest of the months. A further interesting finding is that after including TTD, age variable as a conditional driver of HIE loses its direct effect on decedents’ HIE, but age TTD interaction effect on HIE is still positive and statistically significant.

**Conclusion:** The results confirm that TTD as a proxy of mortality indicator has a considerable effect on decedents’ HIE. The age variable has not directly affected decedents’ HIE but indirectly and through its interaction with TTD has a statistically significant effect on HIE. In addition to age, policy-makers should consider TTD to make better predictions of future HIE.

## Background

**Key Messages**

**Implications for policy makers**

- Time to death (TTD) as a proxy of mortality indicator has a considerable effect on decedents’ hospital inpatient expenditure (HIE). In addition to age, TTD should be considered to make better predictions of future HIE.
- Generally, due to increased mortality and disability at the end of life, a large portion of HIE are incurred in the last years of patient’s life.

**Implications for the public**

This research showed that health status than age per se has a significant effect on hospital inpatient expenditure (HIE) and a considerable portion of people’s health expenditures incur at the late years of life.

One of the important issues in health economics research is the effect of population ageing (increasing the percentage of the population that is 60 years and over) on demand for healthcare and consequently on health expenditure growth. Although ageing is related to high healthcare costs, there is not yet a consensus among researchers about the association between ageing and the growth of healthcare costs.^1^ There are 2 opposing views on this matter. Some analysts and policy-makers, based on a naive approach, believe that healthcare costs are a function of population size, age composition, and age-geder specific healthcare utilization rate.

According to this approach, health expenditure will increase when the population size or the share of elderly groups (as % of the total population) increases.^2^ In contrast, the second approach, proposed by Werblow et al suggests that time to death (TTD) plays a more important role in explaining individual health expenditures, and age does not matter much. Based on this approach, individual healthcare expenditure (HCE) is concentrated in the last years before death and due to high costs of dying, proximity to death can better explain individual healthcare expenses than age.^3^

After this issue, extensive studies were carried out on the relationship of age, proximity to death and health expenditures using micro and macro level data in different countries.^4-9^ However, the results of the studies revealed a large variation in the methods used to collect data, the models used and their estimation and the inclusion of different costs. Subsequently, researchers conducted other empirical studies using different data in the healthcare sector and concluded that the age in some areas of health expenditure remains an important component. According to these studies, age is a major determinant of long-term care expenditures compared to acute hospital care.^10^ Also, taking into account health status indicators such as disability in health costs models reduces the effect of age and TTD.^11^

Given the above-mentioned issues, it is essential to better understand the relationship between age, TTD, and hospital expenditures in order to accurately predict future health spending to meet the challenge of an ageing population in the healthcare system. This is important for developing countries, especially Iran, which will experience significant growth in the ageing population in the coming years.^12-14^ The aim of this study was to determine the effect of age and TTD on decedents’ hospital inpatient expenditure (HIE).

## Methods

###  Data Source 

 As a data source we used a claims database from Iran Health Insurance Organization of Tehran that includes considerable proportion of Tehran city residents and contains information on insured individuals’ HIE. In this study we included HIE of all insured decedents (30 to 90 years old) who died during March 2013 and March 2014 (n = 1018). Then, according to decedants’ date of death, we extracted their last 24 months HIE. In other words, we considered 24 months before their date of death (eg, individual who died in March 11, 2014, we extrected their HIE fom date of March 11, 2012 until March 11, 2014 and so forth). The period of time March 30, 2011 until March 30, 2014 was used to guarantee a full 24 months of observations for decedents (who died during March 2013 and March 2014).

###  Two-Part Model

 Because of the mass point at zero in hospital expenditures (if the illness occurs, then a positive expense will be observed and if not, a zero will be observed) the two-part model provides one approach to account for the mass of zeros and a single index model for such data may not be desirable. In the two-part model, a binary choice model is fit for the probability of observing a positive-versus-zero outcome. Then, conditional on a positive outcome, an appropriate regression model is fit for the positive outcome. Our study’s aim is to estimate a two-part model of HIE as a function of individual-based demographic characteristics and also including TTD expressed in months (HIE in last 24 months of life), in order to investigate the effect of age and TTD on individuals’ HIE.

 In two-part model, a binary choice model (Probit) is fit for the probability of observing a positive-versus-zero outcome (HIE), that can be written as:


(Eq.1) $$P_{r}\left(HIE_{im}>0\left|Xi\right.\right)=\varphi \left(Xi\beta \right)$$

Where HIE_im_ is the HIE of the indivitual *i* in the month *m*; Xi is a vector of explanatory variables, *β *is the corresponding vector of parameters to be estimated, and *φ* represents the standard normal cumulative distribution function. Our first part of model is:


(Eq.2) $$P_{r}\left(HIE_{im}>0\right)=\beta_{0}+\beta_{1}A_{i}+\beta_{2}A_{i}^{2}+\beta_{3}A_{i}^{3}+\beta_{4}S_{i}+\beta_{5}\left(S_{i}.\;A_{i}\right)+\beta_{6}\left(S_{i}.\;A_{i}^{2}\right)+\beta_{7}\left(S_{i}.\;A_{i}^{3}\right)+\beta_{8}MT_{i}+\beta_{9}SI+\sum_{k=0}^{23}{}_{\beta_{10+k}TTD_{ik}}+\beta_{34}\left(TTD_{i}.A_{i}\right)+\beta_{35}Y_{t}+\varepsilon_{i}$$

Where A_i_ is age variable [due to the nonlinear relationship of age with HIE and better specification of the model, we used linear, quadratic, and cubic form of age variable]. S_i_ is a dummy variable for gender (=1 if male, = 0 if female); S_i_. A_i_, S_i_. A_i_^2^ and S_i_. A_i_^3^ are gender age interaction effects, which allows the impact of age to depend on gender; MT is marital status (=1 if married, = 0 if unmarried), SI is dummy variable for supplementary health insurance (=1 if has insurance, = 0 without insurance), TTD_ik_ is the TTD dummy variables of individual_i_ (1 month to 24 months before death), TTD. A_i _is the interaction effect of age TTD, Yt is the calendar-year control (2011 as a base year) and finally ε_im_ is an error term.

Then, conditional on a positive outcome (HIE_im_), an appropriate regression model (GLM, general linear model) is fit for the positive outcome (HIE) that can be written as:

 GLM with a log link:


(Eq.3) $$HIE_{im}=e^{\alpha +\beta x_{i}+\varepsilon_{i}}$$

A GLM with log-link and gamma errors was employed to model HIE, conditional on hospital inpatient care being utilized. The GLM estimation was preferred to the usual ordinaryleast squares estimation of log (HIE_im_) because of the retransformation problems, ie, obtaining a prediction of HIE_im_ from the prediction of log (HIE_im_). Second part of our model (same regressors in first and second parts) is in the following equation:


(Eq.4) $$Ln\left[E\left[HIE_{im}\left|X_{i};>0\right.\right]\right]=\beta_{0}+\beta_{1}A_{i}+\beta_{2}A_{i}^{2}+\beta_{3}A_{i}^{3}+\beta_{4}S_{i}+\beta_{5}\left(S_{i}.\;A_{i}\right)+\beta_{6}\left(S_{i}.\;A_{i}^{2}\right)+\beta_{7}\left(S_{i}.\;A_{i}^{3}\right)+\beta_{8}MT_{i}+\beta_{9}SI+\sum_{k=0}^{23}{}_{\beta_{10+k}TTD_{ik}}+\beta_{34}\left(TTD_{i}.A_{i}\right)+\beta_{35}Y_{t}+\varepsilon_{i}$$

## Results

Table 1 shows the descriptive statistics of the sample. The average age of the dead patients was 65.34. Also, the average TTD in month among decedents was 12. Of the total sample, 55% were men, 74% were married and 76% had supplementary insurance. As shown in the Table 1, mean HIE for the dead patients was $3254. The mean HIE is also shown for different age groups.

**Table 1:** Descriptive Statistics of Sample (n = 1018)

**Variables**	**Mean**	**SD**
Age	65.34	14.689
TTD in months	12	4.391
Share of male	0.55	0.497
Share of married individuals	0.74	0.457
Share of individuals with supplementary insurance	0.76	0.422
Mean HIE of all sample (2011 US$)	$3254.02	8092.662
Mean HIE by age group		
30-34	$1629.92	8385.136
35-39	$1591.88	3954.967
40-44	$1744.54	4254.406
45-49	$2472.06	6101.932
50-54	$2938.79	6292.892
55-59	$3762.49	6932.748
60-64	$4622.53	13 025.861
65-69	$5298.24	10 967.807
70-74	$6198.79	10 419.388
75-79	$6695.37	11 274.534
80-85	$6772.42	11 144.022
90 +	$6934.21	12 411.657
Months with zero HIE	2189	

Abbreviations: TTD, time to death; HIE, hospital inpatient expenditure; SD, standard deviation.

Two part model results are presented in this section. It should be noted that in order to analyze the effect of age and TTD, in first step we estimated the model (Eq. 2 and Eq. 4) without TTD variables (24 dummy variables). In second step we included the TTD variables in the model. The results of these 2 steps are shown in Tables 2 and 3, respectively.

**Table 2:** Two-Part Model Estimations Without TTD

	**Model**								
**Dependent Variable**	**GLM**			**Probit**					
	**Coefficient **	**SE**	***P*** ** Value**	**Coefficient **	**SE**	***P*** ** Value**	**dy/dx**	**SE**	***P *** **> |z|**
Age	-0.226*	0.098	.022	- 0.065**	0.006	.000	- 0.017	0.002	.000
Age^2^/1000	4.804**	1.804	.008	1.858**	0.223	.000	0.237	0.012	.000
Age^3^/1000	-0.028**	0.010	.007	- 0.010**	0.001	.000	- 0.008	0.003	.011
Male	-1.218	2.198	.579	- 0.337	1.056	.750	- 0.095	0.075	.142
Male. Age	0.086	0.126	.491	0.071	0.064	.267			
Male. Age^2^/1000	-1.695	2.315	.464	- 1.684	1.255	.180			
Male. Age^3^/1 000 000	0.010	0.013	.460	0.011	0.007	.156			
Marital status	2.199**	0.125	.000	0.889**	0.050	.000	0.2418	0.016	.000
Supplementary insurance	0.028**	0.004	.000	0.089**	0.031	.005	0.0172	0.006	.006
Yt	0.027	0.026	.301	0.00005	0.0004	.906			
_cons	-45.723	52.675	.385	0.949	24.214	.398			
Observations	22 243			24 432					
R^2^ or pseudo-R^2^	0.342			0.1311					

Abbreviations: TTD, time to death; GLM, general linear model; SE, standard error; _cons, constant; Age^2^, age to power 2; Age^3^, age to power 3. ** Significant at the 99% confidence level; * Significant at the 95% confidence level.

**Table 3:** Two-Part Model Estimations With Time to Death

**Dependent Variable**	**Model**					
	**GLM**			**Probit**		
	**Coefficient**	**SE**	***P*** ** Value**	**Coefficient**	**SE**	***P*** ** Value**
Age	- 0.222	0.198	.213	- 0.040	0.043	.359
Age^2^/1000	0.724	0.485	.131	1.222	0.850	.151
Age^3^/1000	- 0.028	0.021	.164	- 0.006	0.005	.213
Male	- 1.062	2.191	.628	- 0.472	1.454	.746
Male. Age	0.077	0.125	.540	0.079	0.087	.367
Male. Age^2^/1000	-1.501	2.307	.515	- 1.842	1.702	.279
Male. Age^3^/1 000 000	0.008	0.013	.515	0.012	0.010	.248
Marital status	2.199**	0.124	.000	0.892**	0.050	.000
Supplementary insurance	0.153**	0.050	.000	0.089**	0.031	.005
TTD. Age	0.585**	0.066	.000	0.321**	0.044	.000
TTD1	0.850**	0.031	.000	0.986**	0.347	.005
TTD2	0.827**	0.028	.000	0.707**	0.215	.001
TTD3	0. 838**	0.048	.000	0.687**	0.173	.000
TTD4	0.794**	0.025	.000	0.577**	0.168	.001
TTD5	0. 813**	0.021	.000	0.453**	0.158	.004
TTD6	0. 734**	0.032	.000	0.414**	0.150	.006
TTD7	0.717**	0.041	.000	0.393**	0.145	.007
TTD8	0.604**	0.053	.000	0.306*	0.142	.031
TTD9	0.522**	0.183	.007	0.310*	0.139	.025
TTD10	0.510*	0.246	.045	0.309*	0.137	.031
TTD11	0.344	0.251	.156	0.164	0.142	.248
TTD12	0.342	0.294	.202	0.146	1.018	.885
TTD13	0.401	0.311	.172	0.184	1.009	.855
TTD14	0.320	0.330	.250	0.134	1.005	.989
TTD15	0.283	0.332	.272	0.153	1.003	.878
TTD16	0.245	0.328	.313	0.073	1.004	.942
TTD17	0.192	0.451	.357	0.134	1.003	.894
TTD18	0.187	0.420	.359	0.117	1.003	.907
TTD19	0.184	0.435	.364	0.119	1.003	.905
TTD20	0.142	0.401	.375	0.035	1.012	.972
TTD21	0.086	0.509	.392	0.024	1.017	.398
TTD22	0.039	0.558	.390	0.022	1.015	.411
TTD23	0.028	0.563	.398	0.010	1.011	.401
TTD24	0.024	0.506	.384	0.013	1.001	.385
Yt	0.031	0.026	.241	- 0.001	0.016	.951
_cons	-58.075	53.711	.280	2.028	33.269	.951
Observations	22 243			24 432		
R^2^ or pseudo-R^2^	0.1422			0.1316		

Abbreviations: TTD, time to death; GLM, general linear model; SE, standard error; _cons, constant; Age^2^, age to power 2; Age^3^, age to power 3. ** Significant at the 99% confidence level; * Significant at the 95% confidence level.

Results from first and second part of the model (probit and GLM) shows that, the estimated coefficients for age^2^, marital status and supplementary insurance are positive and statistically significant in both parts (Table 2). In other words, both the probability and amount of HIE conditional on any HIE affected by above-mentioned variables. In addition, coefficients of age and age^3^ were statistically significant with negative effects. Gender and its interaction with different forms of age variable were not significant in both step 1 and 2.

Because of nonlinear relationship between age and HIE, age^2^ presumably is a suitable predictor of HIE than age and age,^3^ especially in GLM model that it’s coefficient is even considerable. We also tested jointly significant of age variables coefficients (age, age^2^, age^3^) using Wald test. we tested the hypothesis that the coefficients on 3 age variables jointly are zero. The result of Wald test for the first part (probit model) showed that the null hypothesis was rejected [χ^2^ (3) = 320.07 and Prob > chi2 =.000]. Also, the jointly significant of age coefficients for the second part (GLM) was tested and null hypothesis was rejected [χ^2^ (3) = 490.20 and Prob > chi2 =.000].

However, note that unlike GLM estimations it is difficult to interpret the estimated parameters directly from probit model and only being positive or negative and statistical significance can be infered. Therefore, we estimated marginal effects of statistically significant independent variables (Table 2).

**Figure 1:**
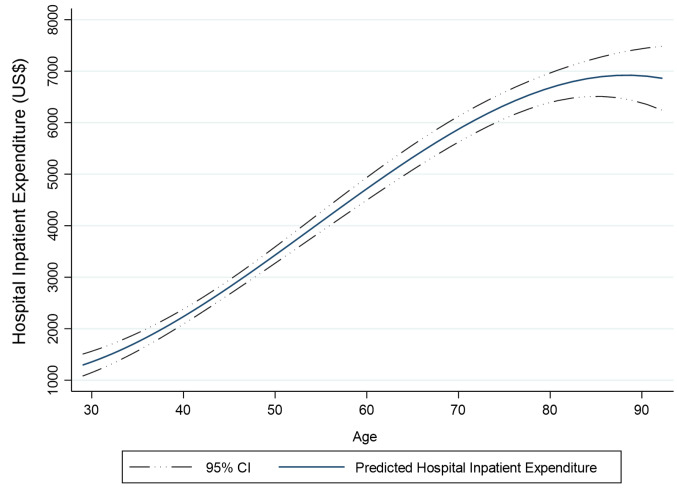
Effect of Age on Predicted Hospital Inpatient Expenditure.

According to the results from marginal effects, a unit increase in age increases the probability of HIE by 0.2 percent points. For the dummy variables including marital status and supplementary insurance it means that the discrete change from the base level (from zero to one) leads to increase 0.241 and 0.017 unit in probability of HIE respectively. Figure 1 demonstrates a relationship between age and predicted HIE.

In second step we ran the model with TTD variables and same dependant variables. Acording to Table 3, after including 24 TTD dummy variables, age variables have lost their direct effects on decedents’ HIE so that their coefficients are statistically insignificant. However, still age TTD interaction effect on HIE is positive and statistically significant. This means that age can affect HIE through interaction with TTD indirectly.

Result of Wald test to investigate jointly significant of age-variables coefficients (age, age^2^, age^3^) in probit model showed that coefficients of age variables jointly are not statistically significant. [χ^2^ (3) = 0.93 and Prob > chi2 = 0.817]. Also, testing the null hypothesis of Wald test for the GLM model indicated that coefficients of age variables jointly are not statistically significant [χ^2^ (3) = 1.34 and Prob > chi2 = 0.718]. Same as previous step, matitial status and supplementary insurance had a positive and statistically significant effect on probability and the amount of HIE conditional on any HIE.

Our results demonstrate that TTD dummy variables (as an indicator of mortality) have statistically significant effect on both the probability and the amount of HIE conditional on any HIE. According to Table 3, in both first and second part of model the effect of the TTD variables was statistically significant from 1 to 10 months before death. From 11 to 24 months before death, the HIE has not been affected by the TTD and was not significant.

**Figure 2:**
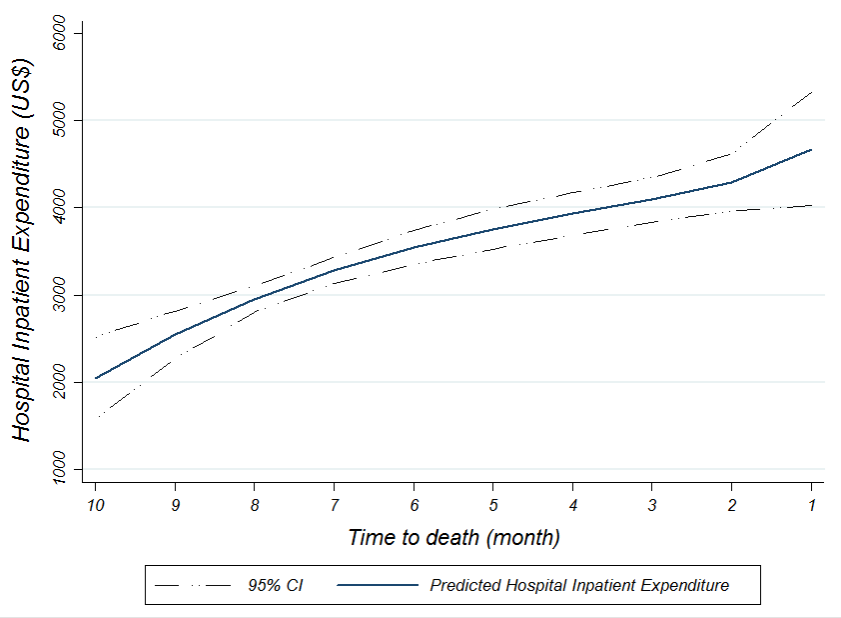
Effect of Time to Death on Predicted Hospital Inpatient Expenditure.

The TTD effect pattern on predicted HIE is shown in Figure 2. Notice that overall trend of the curve has been rising (with decreasing rate) from tenth to second month before death. However, slope of the curve has been steeper from the second month to the first month. From the Figure it is clear that TTD variables had not the same effect on predicted HIE. In other words, one month before death icured more hospital expenditures than other months.

 In general, findings on age and TTD in 2 models (with and without TTD) show that after including TTD variables in the model, age as a conditional predictor of health expenditures loses its influence on HIE.

## Discussion

 Our study’s aim is to estimate a 2 part model of HIE as a function of individual-based demographic characteristics and also including TTD dummy variables, in order to investigate the effect of age and TTD on decedents’ HIE. We used a claims database from Iran Health Insurance Organization of Tehran and included HIE of all insured decedents (30 to 90 years old) who died during March 2013 and March 2014 (n = 1018). In order to analyze the effect of age and TTD, in first step we estimated a 2 part model without TTD variables. In second step we included TTD variables (24 dummy variables) in the model.

Results of the model without TTD dummy variables demonstrate that in both first and second part of the model, age plays an important role in explaining HIE. In other words, when one gets older, both the probability and amount of HIE conditional on any HIE increases. This finding is in line with the traditional role of age that suggests age is associated with healthcare utilization and subsequent expenditures. Moreover, effect of age on amount of HIE conditional on any HIE is even more considerable. That means, age has a more effect on amount of HIE than probability of HIE. Another point is that the effect of age on HIE has not been the same for different ages. As it was shown in Figure 1, the slope of the expedited HIE-age curve has been decreasing and has a concave shape.

Results of the model with TTD dummy variables suggests that TTD dummy variables (TTD1 to TTD10) have statistically significant effect on both the probability and the amount of HIE conditional on any HIE. According to this, near months before death considerably affected both probability and conditional amount of decedents’ HIE than farther months (Table 3 and Figure 2). A further interesting finding is that after including TTD variables, age variable as a conditional driver of HIE loses its direct effect on decedents’ HIE, but age TTD interaction effect on HIE is still positive and statistically significant. This means that age in interaction with TTD and indirectly can affect decedents’ HIE.

In general, because the TTD is a better indicator of the health status than age, it plays a more important role in determining the cost of treatment.^4,15,16^ Moreover, the positive relationship between age and average health expenditures reflects high end-of-life costs and high mortality rates in the elderly.

When comparing our results to previous studies, some similarities and differences can be observed. General results of this study are consistent with those that have used the TTD approach to modeling health expenditures.^7,10,15,17-20^ In these studies, the age has lost its effect on health expenditures after including TTD variables as an indicator of mortality in the model. Zweifel et al in their study and based on the results of the 2 Heckman and two-part models concluded that by including TTD variable in both models, the effect of the age variable on the probability of healthcare utilization has been insignificant.^19,20^ This study showed that the probability of healthcare utilization and spending rates was affected by 1 to 12 months from TTD. Howdon et al suggest that HCE is principally determined by proximity to death rather than age, and that proximity to death is itself a proxy for morbidity.^7^ Recently Wei and Zhou found that when people were near death, their HCEs increased significantly due to the relative concentration of total HCEs before death.^21^ Unlike our results that shows TTD1 to TTD10 have significant effect on HIE, in their study TTD1 and TTD2 had a significant effect on HCE, while the effect of TTD3 was not significant. They concluded that the closer to death the patients were, the higher their HCE. But a different conclusion was reached by Moore et al that showed TTD1 to TTD35 had significant effect one prescribing expenditures.^8^ Some studies suggest that health expenditures in the last year of life are decreasing.^22,23^ Hoover et al in this study concluded that non-Medicare last-year-of-life expenditures were higher and Medicare last-year-of-life expenditures were lower for those dying at older ages. They suggest that some type of care such as hospice care may reduce last-year-of-life expenditures.^23^ Aldridge and Kelley estimated that only 11% of individuals in the highest cost group are in their last year of life. Those with chronic serious illnesses, functional debility, and persistently high costs usually impose high end-of-life expenditures.

Some causes of differences among studies regarding TTD and age can be explained by studies different perspectives in terms of considering type of health expenditures (long term, hospital and medicine expenditures), time period of studies, structure and methodology of analysis. Studies that included hospital and medicine expenditures resulted in more end-of-life costs and significant TTD effects. In return, studies that considered long-term care expenditures concluded moderate end-of-life expenditures.^22,24^

 One concern about the findings of our study was that the focus of the study data on HIE due to lack of access to outpatient and medicine expenditures, so that these expenditures could not be taken into account in analysis and assessing the effects of TTD and age on health expenditures. Despite the fact that the disability index is a more representative indicator of individuals’ health status and their expenditures, considering of such indicators in our study model was not provided due to the lack of available individual’s information. Similar analyses can also be helpful in modeling primary care costs using mortality, morbidity, disability and health status indicators.

## Conclusion

 Based on the results of this study, TTD dummy variables have statistically significant effect on both the probability and the amount of decedents’ HIE conditional on any HIE. In general, TTD as explanatory variable can well illustrate the effect of the mortality and, to some extent morbidity, on HIE than age alone. Also after including TTD variables, age variable as a conditional driver of HIE loses its direct effect on decedents’ HIE, but age TTD interaction effect on decedents’ HIE is still positive and statistically significant. This means that age in interaction with TTD and indirectly can affect decedents’ HIE. Because of increasing mortality and disability high costly treatments, a large portion of HIE is incurred in the last year of life.

## Acknowledgments

 TThe authors would like to make special thanks to all participants for their kind contributions to this project.

## Ethical issues

 This study is a part of PhD thesis and approved by Ethics Committee for Tehran University of Medical Sciences, Tehran, Iran (9021434003).

## Competing interests

 Authors declare that they have no competing interests.

## Authors’ contributions

 Conception and design: VA, AP; Acquisition of data: VA, MK, HH; Analysis and interpretation of data: VA, AP, SES; Drafting of the manuscript: VA; Critical revision of the manuscript for important intellectual content: AP; Statistical analysis: VA, HH; Supervision: AP.

## Authors’ affiliations

^1^Health Management and Economics Research Center, Iran University of Medical Sciences, Tehran, Iran. ^2^Department of Health Education and Promotion, School of Public Health, Tehran University of Medical Sciences, Tehran, Iran. ^3^Department of Demography, Social Science Faculty, Tehran University, Tehran, Iran. ^4^Department of Economic Sciences, School of Management and Economics, Tarbiat Modares University, Tehran, Iran. ^5^Department of Health Management and Economics, School of Public Health, Tehran University of Medical Sciences, Tehran, Iran.
